# Polyamine Catabolism and Its Role in Renal Injury and Fibrosis in Mice Subjected to Repeated Low-Dose Cisplatin Treatment

**DOI:** 10.3390/biomedicines12030640

**Published:** 2024-03-13

**Authors:** Kamyar Zahedi, Sharon Barone, Marybeth Brooks, Tracy Murray Stewart, Jackson R. Foley, Ashley Nwafor, Robert A. Casero, Manoocher Soleimani

**Affiliations:** 1 Division of Nephrology, Department of Medicine, University of New Mexico Health Sciences Center, Albuquerque, NM 87131, USA; 2 Research Services, New Mexico Veterans Health Care Center, Albuquerque, NM 87108, USA; 3 The Sidney Kimmel Comprehensive Cancer Center, Johns Hopkins University School of Medicine, Baltimore, MD 21231, USA

**Keywords:** cisplatin, nephrotoxicity, chronic kidney injury, fibrosis, polyamine, polyamine catabolism

## Abstract

Cisplatin, a chemotherapeutic agent, can cause nephrotoxic and ototoxic injuries. Using a mouse model of repeated low dose cisplatin (RLDC), we compared the kidneys of cisplatin- and vehicle-treated mice on days 3 (early injury phase) and 35 (late injury/recovery phase) after the final treatment. RNA-seq analyses revealed increases in the expression of markers of kidney injury (e.g., lipocalin 2 and kidney injury molecule 1) and fibrosis (e.g., collagen 1, fibronectin, and vimentin 1) in RLDC mice. In addition, we observed increased expression of polyamine catabolic enzymes (spermidine/spermine N^1^-acetyltransferase, *Sat1*, and spermine oxidase, *Smox*) and decreased expression of ornithine decarboxylase (*Odc1*), a rate-limiting enzyme in polyamine synthesis in mice subjected to RLDC. Upon confirmation of the RNA-seq results, we tested the hypothesis that enhanced polyamine catabolism contributes to the onset of renal injury and development of fibrosis. To test our hypothesis, we compared the severity of RLDC-induced renal injury and fibrosis in wildtype (WT), *Sat1*-KO, and *Smox*-KO mice. Our results suggest that the ablation of polyamine catabolic enzymes reduces the severity of renal injury and that modulation of the activity of these enzymes may protect against kidney damage and fibrosis caused by cisplatin treatment.

## 1. Introduction

Cisplatin is a chemotherapeutic agent that is used to treat a variety of solid tumors [1]. Its antitumor activity is mediated through the formation of stable DNA adducts that result in cell cycle arrest and death [2]. Cisplatin also has off-target adverse effects and can cause nephrotoxic and ototoxic injuries [3,4]. In more than 25% of patients, the toxic off-target effects of cisplatin lead to discontinuation of treatment [3,5].

**Scheme 1:**
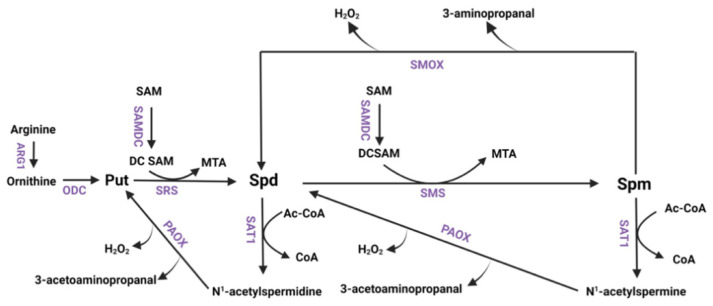
Polyamine metabolic pathway.

Polyamines, spermine (Spm), and spermidine (Spd), are naturally occurring polycationic alkylamines. They are important regulators of DNA structure, DNA-protein and protein–protein interactions, as well as free radical scavengers [6,7,8,9,10,11]. As such, they are important in the maintenance of genomic integrity and the regulation of cell growth and viability [7,8,9,10]. Cellular polyamine levels are controlled via their export, import, synthesis, and degradation [12,13,14,15]. Polyamine synthesis is initiated by the decarboxylation of ornithine to form putrescine (Put), a reaction that is mediated by ornithine decarboxylase1 (ODC1), the first rate-limiting enzyme in polyamine synthesis. This is followed by the sequential enzymatic addition of aminopropyl groups to Put and Spd by spermidine synthase (SRM) and spermine synthase (SMS), respectively, to generate Spd and Spm [14,16,17]. Polyamine catabolism can result in decreased intracellular polyamines through multiple mechanisms [18,19,20]. Spm and Spd are acetylated by SAT1 and can either be excreted from the cell or serve as substrates for acetylpolyamine oxidase (PAOX) [12,15]. Further, Spm can be directly converted to Spd by spermine oxidase (SMOX), a highly inducible enzyme [21,22]. Oxidation of SAT1-generated *N*^1^-acetyl-Spm and -Spd generated by PAOX and Spm by SMOX leads to the generation of cytotoxic compounds (e.g., H_2_O_2_ and reactive aldehydes) [21]. An outline of polyamine metabolism is presented in Scheme 1. Because of the high cellular content of polyamines, which is in the mM range, substantial quantities of H_2_O_2_ and reactive aldehydes such as 3-aminopropanal, 3-acetoaminopropanal, and acrolein can be produced as a result of their catabolism [23]. These cytotoxic products can lead to nucleic acid and protein damage and disrupt the integrity of lysosomal and mitochondrial membranes [24,25,26,27]. The adverse effect of enhanced polyamine catabolism has also been demonstrated in vitro, where overexpression of SAT1 in cultured cells leads to increased SMOX expression, alterations in polyamine homeostasis, DNA damage, mitochondrial dysfunction, groWTh arrest, and apoptosis [28,29,30,31,32].

Catabolism of polyamines is enhanced in the kidney, brain, liver, stomach, colon, and heart in response to a variety of injuries [33,34,35,36,37]. In addition, the expression of polyamine catabolic enzymes increases and is associated with remote organ dysfunction following an initial injury [38]. Increased polyamine catabolism can cause tissue/organ damage consequent to decreased levels of radical-scavenging natural polyamines, the generation of reactive oxygen molecules (e.g., H_2_O_2_), and/or the production of reactive aldehydes (e.g., aminoaldehydes and acrolein) [33,39,40,41,42]. In mice, the ablation or inhibition of the *Sat1* and *Smox* genes or neutralization of their toxic products reduces the severity of tissue damage caused by various insults [31,36,42,43].

A single high dose of cisplatin (20–30 mg/kg) causes acute nephrotoxic injury in rodents [44,45]. The catabolism of polyamines is up-regulated in such acute cisplatin nephrotoxicities in both rats and mice [37,42]. The ablation of genes encoding polyamine catabolic enzymes, *Sat1* and *Smox*, or the neutralization of their by-products, reduced the severity of acute cisplatin nephrotoxic injury [42]. The above observations indicate that the up-regulation of polyamine catabolism is important in the mediation of acute cisplatin nephrotoxicity [37,42]. Although high-dose cisplatin models clearly demonstrate the nephrotoxicity of cisplatin, they do not represent the multidose course of cisplatin treatment in cancer patients [46]. A recent model of low-dose cisplatin injury, which more closely resembles the course of treatment in cancer patients, has been developed, which indicates that low doses of cisplatin provided over a prolonged period lead to renal tubular injury, fibrosis, and chronic kidney dysfunction [46,47,48].

We propose that the anti-neoplastic effects of cisplatin (stable DNA-adduct formation) are disparate from much of its general toxic effects and that treatments that reduce the toxic effects of this drug while minimally affecting its anti-tumor activity can enhance the tolerance and effectiveness of cisplatin treatment. We hypothesize that the disruption of polyamine catabolism will reduce the chronic kidney damage associated with long-term cisplatin treatment. To test this hypothesis, we utilized the multiple low-dose cisplatin (RLDC) model of kidney injury and fibrosis in mice, developed by Sharp et al. [47], to compare the severity of acute renal injury and fibrotic response in WT, *Sat1*-KO and *Smox*-KO mice.

## 2. Materials and Methods

### 2.1. Reagents

The chemicals and reagents used in these studies were purchased from Sigma–Aldrich (St. Louis, MO, USA), unless otherwise indicated. Oligonucleotides were purchased from ThermoFisher Scientific (Carlsbad, CA, USA). The following antibodies were used in this study: rabbit anti-Smox (Proteintech; Rosemont, IL, USA), rabbit anti-SAT1 (Proteintech), and rabbit anti-F4/80 (Abcam; Waltham, MA, USA). The secondary antibodies were purchased from ThermoFisher Scientific.

### 2.2. Generation and Genotyping of Sat1- and Smox-KO Mice

Spermidine/spermine-N1-acetyltransferase (SSAT)-deficient (*Sat1*-KO) mice were the kind gift of Dr. Carl W. Porter. Their generation and genotyping protocol have been previously described [49,50]. SMOX-deficient (*Smox*-KO) mice were generated by the elimination of exons 4–6 through homologous recombination and were genotyped, as previously described [42]. All animals used in these studies were bred onto the C57BL/6J background.

### 2.3. Mouse RLCD

All animal studies were designed using ARRIVE guidelines and were approved by the University of New Mexico and University of Cincinnati Institutional Animal Care and Use Committees (IACUC, protocol number 04020901). WT and knockout mice (*n* = 8–10/treatment group) were given a single weekly intraperitoneal (I.P.) injection of vehicle (saline) or cisplatin (7 mg/kg) for four consecutive weeks. Animals were euthanized by an overdose of Euthazol (150 µL of 390 mg sodium pentobarbital and 50 mg phenytoin/100 mL) and processed to obtain the needed specimens, including serum for the measurement of BUN and creatinine levels, kidneys for the extraction of RNA and protein, as well as measurements of polyamines and polyamine pathway enzymes. Kidney samples used for histology (H&E stain), immunofluorescence microscopy, and immunohistochemistry were fixed in paraformaldehyde and preserved in 70% ethanol. Renal fibrosis was assessed using Masson Trichrome Stain. 

### 2.4. Assessment of Renal Function

Serum creatinine and BUN levels were measured using a commercially available kit (Bioassay Systems, Hayward, CA, USA) following the manufacturer’s instructions.

### 2.5. Histopathology, Immunofluorescence Microscopy, and Immunohistochemical Examination of Kidneys

Paraformaldehyde-fixed, ethanol-preserved kidney samples were paraffin-embedded, and 5-µm sections were cut and used for further studies. The severity of renal damage was determined by examining the cortical and corticomedullary regions of H&E-stained kidney sections for tubular damage and cast formation. Infiltration of macrophages was determined by immunohistochemical (IHC) staining for F4/80, utilizing an IHC kit (Vector Labs; Newark, CA, USA). Staining was performed following the manufacturer’s protocol. For immunofluorescence microscopic analysis of kidneys, the sections were deparaffinized in xylene, rehydrated, and boiled for 20 min in R-Universal Epitope Recovery Buffer (Electron Microscopy Sciences; Hatfield, PA, USA) for antigen unmasking. Sections were blocked in PBS containing 1% BSA, 0.2% powdered skim milk, and 0.3% Triton X-100 for at least 60 min at room temperature before incubation with primary antibodies overnight at 4 °C. After washing, tissue sections were incubated with the appropriate secondary antibodies overnight at 4 °C. 

### 2.6. Measurement of Kidney Polyamine and ODC Levels

Cellular polyamine content was determined, as previously described [51,52]. Briefly, perchloric acid extracts of the harvested kidneys were dansylated, and chromatographs were resolved by reverse-phase high-performance liquid chromatography with an increasing acetonitrile/H_2_O gradient. ODC activity was measured following a previously described protocol [53,54].

### 2.7. RNA Extraction and Northern Blot Analysis

RNA was extracted using Tri-Reagent (MRC; Cincinnati, OH, USA) and subjected to northern blot analysis.

### 2.8. RNA-Seq Analysis

RNA-seq studies were performed by Novogene Bioinformatics Technology Co., Ltd. (Sacramento, CA, USA). Briefly, total RNA was isolated from the kidneys and subjected to quality control analysis, using an Agilent 2100 Bioanalyzer with RNA 6000 Nano Kits (Agilent; Colorado Springs, CO, USA). After poly A selection, the samples were fragmented and reverse-transcribed to generate complementary DNA for sequencing. Libraries were sequenced on the HiSeqTM 2500 system (Illumina; San Diego, CA, USA). Clean reads were aligned with a mouse reference genome using Hisat2 v2.0.4. Gene expression levels were estimated using fragments per kilobase of transcript per million mapped fragments (FPKM) by HTSeq v0.9.1.

### 2.9. Statistical Analysis

The statistical differences between mean values +/− SD of multiple samples were determined using a one-tailed unpaired Student’s *t*-test. A *p-*value of less than 0.05 was considered statistically significant. 

## 3. Results

### 3.1. RLDC Treatment Leads to Early and Persistent Renal Injury

**Figure 1:**
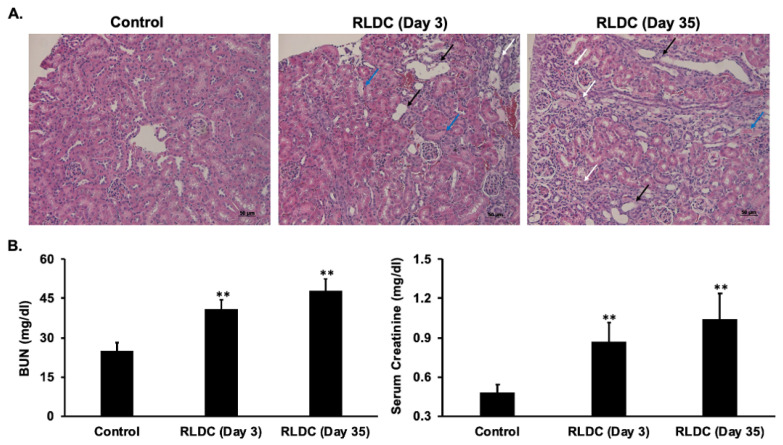
RLDC treatment leads to early and persistent renal injury. Kidney histology and renal function of mice from controls (vehicle-treated) and mice subjected to RLDC (days 3 and 35 post-last injection) were examined. (**A**) The kidney samples stained with H&E indicate the presence of tubular dilatation (black arrows), cast formation (blue arrows). and potential fibrosis (white arrows) in the kidneys of mice subjected to RLDC. (**B**) BUN (left panel) and serum creatinine (right panel) levels were significantly (*p* < 0.01) elevated in mice subjected to RLDC. ** Denotes a *p* < 0.01.

**Figure 2:**
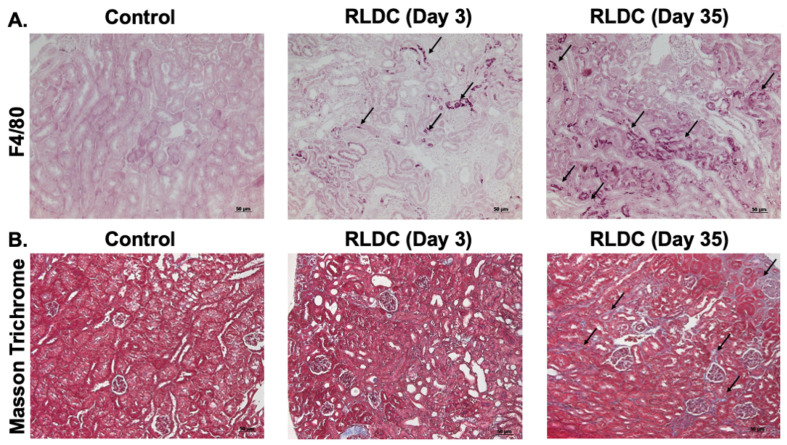
RLDC treatment is associated with renal fibrosis and macrophage cell infiltration. Kidney sections of mice from control (vehicle-treated) and RLDC groups (days 3 and 35 post-last injection) were examined for macrophage infiltration and interstitial fibrosis. (**A**) Immunohistochemical staining with antibodies against F4/80 (ADGRE1) revealed increased infiltration of macrophages in response to RLDC treatment (middle and right panels; black arrows). (**B**) Masson Trichrome staining of renal sections from control and treated mice revealed increased renal fibrosis in mice on day 35 after the final cisplatin treatment (right panel; black arrows).

Mice subjected to RLDC develop injuries to the renal parenchyma (e.g., tubular epithelial damage, cast formation, and fibrosis). These injuries lead to deficits in renal function, which are reflected in significant increases in serum creatinine and blood urea nitrogen (BUN) levels (Figure 1A,B). The renal injury was associated with increased macrophage infiltration, which was apparent as early as three days after the fourth and final cisplatin administration and persisted through the course of the studies (35 days after the final cisplatin administration, Figure 2A). Mice subjected to RLDC also developed interstitial fibrosis 35 days after the final cisplatin administration (Figure 2B).

### 3.2. Comparison of Transcriptomes of Control Mice and Mice Subjected to RLDC

RNA was extracted from the kidneys of control mice and mice exposed to RLDC on days 3 and 35 after final treatment and subjected to RNA-seq analysis. RNA-seq analyses were repeated twice for day 3 (*n* = 5 total biological replicates) and day 35 (*n* = 7 total biological replicates). In the kidneys of treated mice vs. control mice, a total of 2644 transcripts from day 3 and 2218 transcripts from day 35 were significantly down-regulated (log_2_ fold < −0.3 and padj < 0.05) (e.g., *Slc22a7*, *Slc22a28*, kidney androgen-regulated protein, *Lipo2*, and lipoprotein lipase; Supplementary datasets S1–S4). The expression of mitochondrial transcription factor A (*Tfam*), a fibrosis-associated protein [55], was down-regulated on both days 3 and 35, indicating the disruption of mitochondrial function and initiation of fibrotic changes in the kidneys. Comparison of differentially expressed transcripts (DETs) also denoted that 2879 (day 3) and 2314 (day 35) mRNAs were significantly up-regulated (log_2_ fold > 0.3 and padj < 0.05) in RLDC-treated kidney transcriptomes (Supplementary datasets S1–S4). These DETs included markers of kidney injury (e.g., lipocalin 2 and kidney injury molecule 1), inflammation (e.g., chemokines, interleukins, and tumor necrosis factor), inflammatory cell infiltration (e.g., *Adgre1* and *Mki67*), and fibrosis (e.g., collagen 1, fibronectin, and vimentin 1).

**Figure 3:**
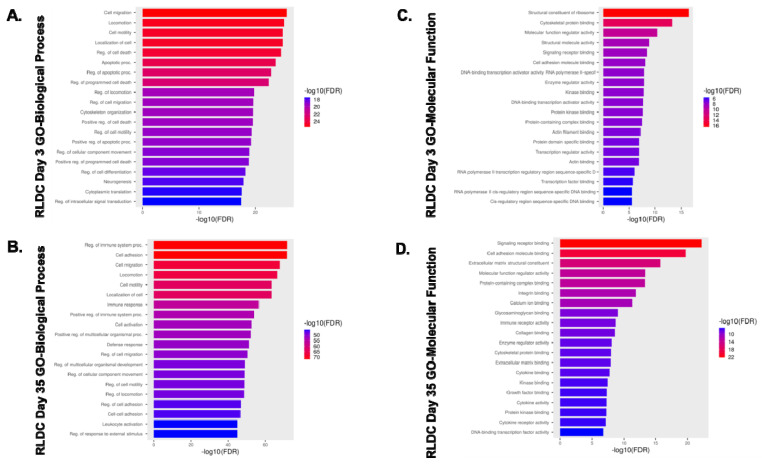
GO enrichment analysis of significantly up-regulated DETs. Using the Shiny GO web application (http://bioinformatics.sdstate.edu/go accessed on 14 August 2023), the significantly up-regulated DETs were subjected to enrichment analysis. (**A**,**B**) Enrichment bar plots for GO-Biological Process. (**C**,**D**) Enrichment bar plots for GO-Molecular Function. Examination of day 3 DETs identified more than 1000 biological process and 143 molecular function terms that were significantly (FDR < 0.05) over-represented in mice exposed to RLDC. Examination of day 35 results showed that over 1000 biological process and 206 molecular function terms were significantly over-represented in RLDC compared to control mice. The complete listing of enriched GO pathways can be found in Supplementary datasets S5–S8.

**Figure 4:**
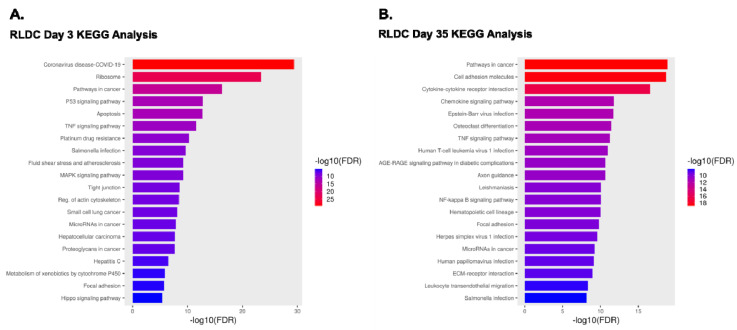
KEGG enrichment analysis of significantly up-regulated DETs. Using the Shiny GO web application, the significantly up-regulated DETs were subjected to KEGG enrichment analysis. (**A**,**B**) KEGG enrichment bar plots for mice on days 3 and 35 after the final dose of cisplatin was administered. The pathways related to programmed cell death (e.g., P53 signaling and apoptosis), TNF and cytokine signaling, as well as cellular interactions (e.g., focal adhesion and ECM-receptor interaction) were significantly enriched in the kidneys of mice subjected to RLDC on days 3 and 35. The complete list of enriched KEGG pathways can be found in Supplementary datasets S9 and S10.

GO enrichment analysis of the significantly up-regulated transcripts in day 3 samples revealed a total of 1000+ biological process and 143 molecular function terms, significantly (FDR < 0.05) over-represented in day 3 RLDC mice (Figure 3A,B and Supplementary datasets S5 and S6). Examination of day 35 results showed that 1000+ biological process and 206 molecular function terms were significantly over-represented in RLDC compared to control mice (Figure 3C,D and Supplementary datasets S7 and S8). In addition, KEGG enrichment analysis of mice subjected to RLDC treatment on days 3 and 35 indicated enrichment for 120 and 155 pathways, respectively. The pathways related to programmed cell death (e.g., P53 signaling and apoptosis), TNF and cytokine signaling, as well as cellular interactions (e.g., focal adhesion and ECM-receptor interaction) were significantly enriched in the kidneys of RLDC mice (Figure 4A,B and Supplementary datasets S9 and S10).

### 3.3. Examination of the Expression of Polyamine Pathway Enzymes in RLDC Mice

**Figure 5:**
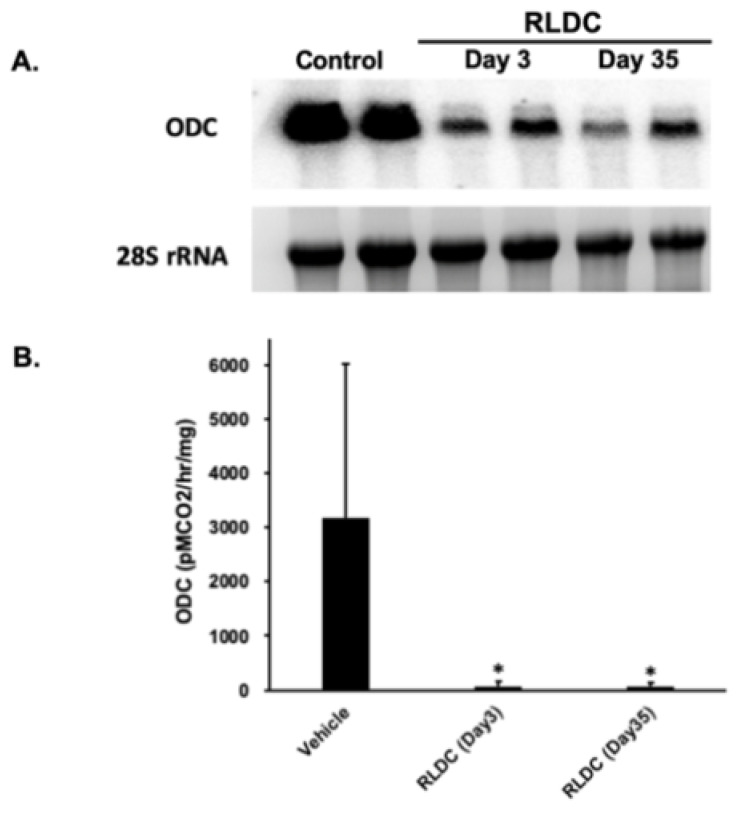
Examination of the expression of *Odc* and its enzymatic activity in the kidneys of mice subjected to RLDC treatment. The expression of *Odc* (**A**) and its activity (**B**) were determined in the kidneys of control mice and mice subjected to RLDC. Our results indicate that the expression of *Odc* mRNA and enzymatic activity of ODC were significantly reduced in animals subjected to RLDC treatment. * Denotes a *p*-value of <0.05. The entirety of the northern blot analysis results used in this figure are shown in Supplementary Figure S2.

The examination of RNA-seq results for the expression of enzymes involved in polyamine synthesis showed that while the renal expression of spermidine synthase (*Srm*) was not altered, the expression of spermine synthase (*Sms*) was significantly reduced in day 3 RLDC samples. The expression of *Odc1*, the first rate-limiting enzyme in polyamine synthesis, was significantly decreased in the kidneys of RLDC mice during the early (day 3) and late (day 35) injury phases by log_2_ folds of −2.1 and −1.4, respectively (Supplementary datasets S1 and S2). The reduced expression of *Odc* was confirmed by northern blot analysis and through comparing its enzymatic activity in the kidneys of RLDC and control mice (Figure 5A,B).

**Figure 6:**
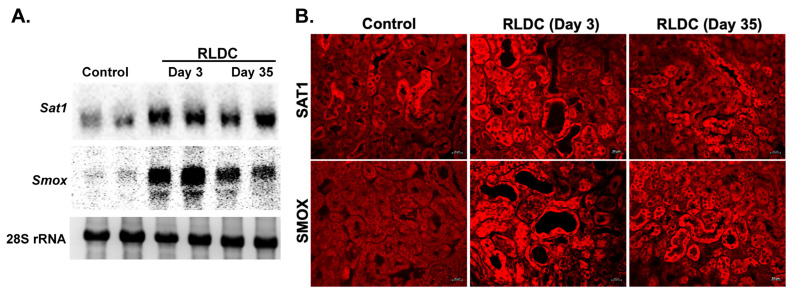
Examination of the expression and localization of Sat1 and Smox in the kidneys of mice subjected to RLDC treatment. The expression levels and renal localization of polyamine catabolic enzymes, SAT1 and SMOX, were examined by northern blot analysis and immunofluorescence microscopy. (**A**) The expression of *Sat1* and *Smox* transcripts in the kidneys of control and RLDC-treated mice were compared. The results indicate that the expression of both transcripts increased in response to RLDC treatment. (**B**) Immunofluorescence microscopic analysis of the kidneys of control mice and mice subjected to RLDC indicated that the expression of SAT1 and SMOX increases in the renal tubules of cisplatin-treated mice. The entirety of the northern blot results used in this figure are shown in Supplementary Figure S2.

RNA-seq results also indicated that the expression of transcript coding for the polyamine catabolic enzymes, *Sat1* and *Smox,* increased significantly (padj < 0.05) by log_2_ folds of 0.94 and 1.7, respectively, in the early injury phase (day 3) of RLDC. Confirmatory northern blot analyses validated the increased expression of *Sat1* and *Smox* in the kidneys of RLDC mice in the early injury phase (day 3) as well as the later recovery/fibrosis phase (day 35) samples (Figure 6A). Next, to determine the sites of expression of SAT1 and SMOX, we analyzed their renal expression by immunofluorescence microscopy. Enhanced expression of SAT1 and SMOX was detected on days 3 and 35 in the tubules of the corticomedullary region of the kidneys of mice subjected to RLDC (Figure 6B).

### 3.4. Comparison of Renal Polyamine Levels in Control and RLDC Mice

**Figure 7:**
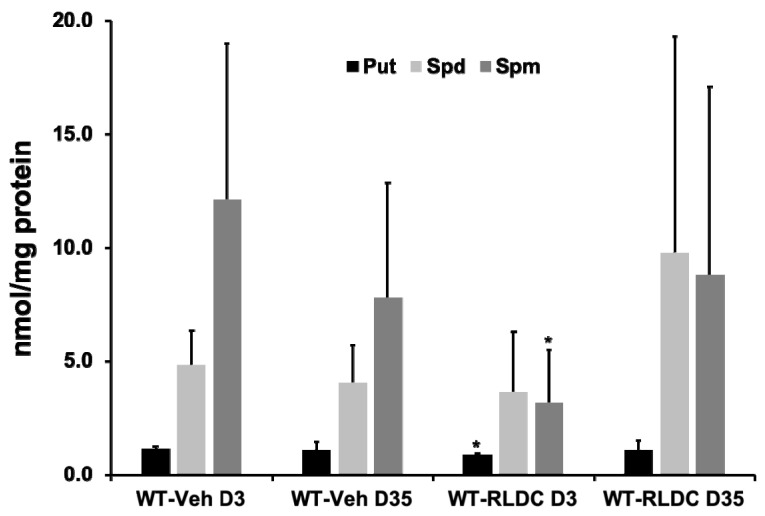
Comparison of renal polyamine levels in control mice and mice subjected to RLDC treatment. Polyamine levels (nmol/mg protein) were determined in the kidneys of control WT mice and WT mice subjected to RLDC treatment. The results suggest that renal Put and Spm are significantly different on day 3 post-RLDC compared to vehicle controls (Veh). By day 35, after the completion of treatment, the renal polyamine levels are not significantly different in animals that were subjected to Veh vs. RLDC treatment. The (*) denotes a *p*-value < 0.05 compared to time-matched control animals.

Next, we compared the polyamine levels in the kidneys of mice subjected to RLDC to those of vehicle-treated control mice. Our results revealed that RLDC treatment leads to significant changes in the renal content of Put and Spm in the early (day 3) injury phase (Figure 7). The renal polyamine content of kidneys on day 35 did not significantly differ in RLDC and control (vehicle-treated) mice.

### 3.5. Ablation of Sat1 or Smox Reduces the Severity of RLDC-Induced Renal Injury and Fibrosis

Enhanced polyamine catabolism is important in the mediation of tissue damage in a variety of injuries [11,36,43,56,57,58]. To examine the role of polyamine catabolism in the mediation of renal injury caused by RLDC, we compared the severity of kidney damage, polyamine levels, the onset of renal fibrosis, and kidney function in WT compared to *Sat1*-KO and *Smox*-KO mice. 

**Figure 8:**
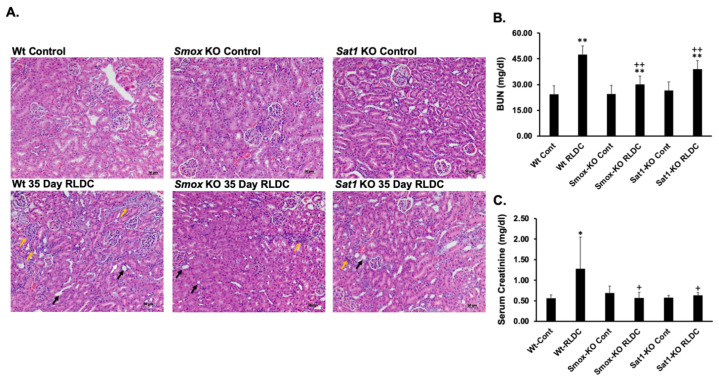
The ablation of *Sat1* or *Smox* genes reduces the severity of renal injury and dysfunction resulting from RLDC administration. The renal histology and function of WT, *Smox*-KO, and *Sat1*-KO mice treated with vehicle or subjected to RLDC treatment were compared. (**A**) Kidney sections from wild-type, *Smox*-KO, and *Sat1*-KO mice treated with vehicle or RLDC (Day 35) were stained with H&E. The comparison of renal histology shows the presence of renal injury, as marked by tubular dilatation (black arrows) and areas of fibrosis (orange arrows). (**B**,**C**) The BUN (upper panel) and serum creatinine (lower panel) levels were significantly elevated in wild-type but not *Smox*-KO and *Sat1*-KO mice subjected to RLDC. * Denotes a *p*-value < 0.05 when control and RLDC samples are compared. ** Denotes a *p*-value of <0.01 when control and RLDC samples are compared. ^+^ Denotes a *p*-value < 0.05 when WT and KO RLDC samples are compared. ^++^ Denotes a *p*-value < 0.01 when WT and KO RLDC samples are compared.

The examination of H&E-stained slides revealed that while WT mice had significant renal injuries 35 days post-RLDC, the kidneys of time-matched *Sat1*-KO and *Smox-*KO mice were protected against renal damage caused by RLDC treatment (Figure 8A). This observation was further supported by the preservation of renal function in *Sat1*-KO and *Smox*-KO compared to WT mice (Figure 8B).

Next, we analyzed the renal polyamine levels in WT, *Sat1*-KO, and *Smox*-KO mice treated with saline or subjected to RLDC treatment (Supplementary Figure S1). Our results suggest that the renal content of Spm was the most significantly changed of all three molecules. The *Smox*-KO mice had significantly higher kidney Spm levels than the WT mice under either baseline (control) conditions or after RLDC treatment (day 35 post-final injection). 

**Figure 9:**
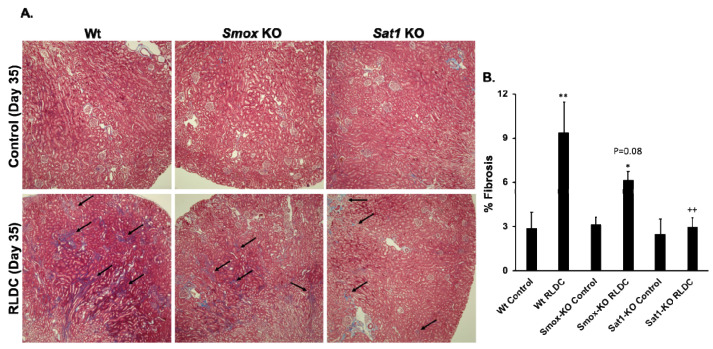
The effect of the ablation of *Sat1* or *Smox* on the severity of renal fibrosis in response to RLDC treatment. The extent of interstitial fibrosis in the kidneys of WT, *Smox*-KO, and *Sat1*-KO mice subjected to RLDC treatment was examined. (**A**) Masson Trichrome staining of kidney sections from wild-type, *Smox*-KO, and *Sat1*-KO mice subjected to RLDC shows the development of interstitial fibrosis (black arrows). (**B**) The extent of renal interstitial fibrosis was quantitated using Image J software (release 1.53K). The results suggest that the ablation of *Smox* and *Sat1* genes protects against the development of fibrosis after RLDC treatment. * Denotes a *p*-value < 0.05 when the extent of fibrosis in the kidneys of RLDC treated vs. control mice were compared. ** Denotes a *p*-value < 0.01 when the extent of fibrosis in the kidneys of RLDC treated vs. control mice were compared. ^++^ Denotes a *p*-value < 0.01 when the extent of fibrosis in the kidneys of WT and knockout mice subjected to RLDC treatment were compared. *p* = 0.08 denotes the *p*-value obtained upon comparison of fibrotic response in WT vs. *Smox*-KO mice after RLDC.

Comparing the severity of renal fibrosis using Masson Trichrome staining, it was demonstrated that *Sat1*-KO and *Smox*-KO mice had significantly reduced levels of fibrosis compared to their WT counterparts (Figure 9A,B). 

## 4. Discussion

Platinum compounds (e.g., cisplatin and its derivatives) are highly effective chemotherapeutic agents that are used for the treatment of many solid tumors [1,3]. The use of these compounds is associated with off-target toxic effects, such as ototoxicity and nephrotoxicity, which limit their effectiveness [3,4,5]. The nephrotoxicity of cisplatin has been experimentally documented in rodents, but a limitation of these studies is the utilization of a model of single high-dose acute toxicity rather than a more clinically relevant multidose chronic injury [46,47]. The RLDC mouse model of cisplatin toxicity closely mimics the multidose cisplatin chemotherapy approach used in the treatment of cancer patients [46,47]. In these studies, we used RNA-seq and bioinformatics analysis to compare the renal transcriptome alterations in the early (day 3 after the completion of cisplatin treatment) and late phases (day 35 after the completion of cisplatin treatment) of RLDC to that of saline (vehicle)-treated control mice. RLDC treatment led to renal tubular damage, renal fibrosis, and renal dysfunction (Figure 1 and Figure 2). The transcriptome changes in RLDC-treated mice included increases in the expression of injury markers (e.g., *Lcn2* and *Kim1*), inflammatory mediators (e.g., *Ccl2*, *Ccl20*, *Cxcl2*, and *Tnfα*), and profibrotic factors (e.g., fibronectin, collagen 1 components, vimentin, and transforming groWTh factor β; for additional information, please refer to Supplementary datasets S1–S4). KEGG analysis identified pathways associated with inflammation (e.g., TNF-α, IL17, cytokines, JAK/STAT, as well as NFκB signaling), apoptosis, innate immune response, and recovery (e.g., tight junction, focal adhesion, and VEGF signaling), which were significantly (FDR < 0.05) enriched in the kidneys of mice subjected to RLDC compared to their control counterparts (Figure 4A,B and Supplementary datasets S9 and S10). The changes in gene expression and pathway analysis correlated with renal parenchymal damage, loss of renal function, and fibrosis (Figure 1 and Figure 2). These results suggest the active ongoing processes of tissue injury at both days 3 and 35 post-RLDC and repair/fibrosis at 35 days post-RLDC time points. Similar injury dynamics were documented in snRNA-seq studies examining the gene expression in the late phase (nine weeks after the completion of cisplatin treatment) of RLDC [59]. The assessment of the macrophage marker, F4/80 (*Adgre1*), revealed that its levels are significantly increased in the late phase of RLDC. The changes in F4/80 levels were further confirmed by immunohistochemistry (Figure 2A). In RLDC-treated mouse kidneys, F4/80-stained macrophages were visible at day 3 and were significantly elevated at day 35 (Figure 2B). The change in F4/80 expression correlated with the development of fibrosis in the late/recovery phase of RLDC (Figure 2A). The association of increased F4/80 expression by macrophages and the development of renal fibrosis in RLDC have been documented by Sears et al., who demonstrated that the depletion of high F4/80-expressing M2 macrophages attenuated the development of renal fibrosis [60]. Our results are congruent with these findings and support the potential importance of resident kidney macrophages in the fibrotic process. 

Further analysis of RNA-seq results indicated that polyamine metabolism is significantly altered in mice subjected to RLDC (Supplementary datasets S1–S4). Data revealed that the expression of *Odc1*, the first rate-limiting enzyme in polyamine synthesis, is down-regulated in the kidneys of animals that were subjected to RLDC on both days 3 and 35 post-treatment. On the other hand, the renal expression of *Sms* is only reduced in day 3 RLDC mice. The RNA-seq data further indicated that the expression of transcript coding for the polyamine catabolic enzymes, *Sat1* and *Smox*, were significantly increased in day 3 samples. The accuracy of the RNA-seq results were further examined by northern blot analysis and immunofluorescence microscopic examination of RNA and kidney sections from control and RLDC mice, respectively. The aforementioned studies indicate that the mRNA levels of both *Sat1* and *Smox* transcripts, as well as the expression of SAT1 and SMOX proteins, were significantly elevated on both days 3 and 35 after the completion of RLDC treatment (Figure 6).

The examination of kidney polyamine levels indicated that while the renal contents of Put and Spm are significantly affected in RLDC animals in the early period of tissue injury (day 3), polyamine levels were not significantly altered when control and RLDC-treated WT mice were compared on day 35 post-treatment. Additional studies were performed to determine the effect of RLDC on tissue polyamine levels in WT, *Sat1*-KO, and *Smox*-KO mice. The examination of Spm levels in the kidneys of *Smox*-KO mice subjected to RLDC revealed that compared to WT mice, the Smox-KO mice maintained significantly higher renal Spm levels under normal conditions and after RLDC treatment (Supplementary Figure S1). These results suggest that in WT mice, the disturbance in kidney polyamine levels, especially Put and Spm, may be important in the mediation of RLDC-induced kidney injury in early time points. The elevated Spm levels observed in the kidneys of *Smox*-KO mice suggests that Spm may function to reduce the severity of renal injury in this strain. The potential protective effect of Spm is supported by studies that describe its role as an oxygen scavenger and a molecule with anti-inflammatory functions [11,17,61,62]. The role of toxic by-products of polyamine degradation, such as H_2_O_2_ and 3-aminopropanal, which contribute to acute cisplatin nephrotoxicity, may also be involved in the mediation of RLDC-induced tissue damage. This possibility will be pursued, since previous studies have demonstrated the maladaptive role of the toxic products of polyamine degradation in the mediation of tissue injury [40,42,63]. 

The early induction of polyamine catabolic enzymes suggests that they are important in the mediation of renal injury in the early/injury phases of RLDC, while their continued expression suggest the presence of their ongoing role in the chronic response to RLDC (Figure 6). The adverse role of enhanced polyamine catabolism in the mediation of kidney injury in RLDC is further supported by the reduction in renal injury, better preservation of renal function, and reduced renal fibrosis in *Sat1*-KO and *Smox-*KO compared to WT mice (Figure 8 and Figure 9). The extent of damage to the tubular epithelium is an important determinant of the development of renal fibrosis [64,65,66]. Considering the maladaptive role of enhanced SAT1 and SMOX in the induction of renal tubular injury [67], the preservation of renal function, decreased damage to the tubular epithelium, and reduced fibrosis in *Sat1-*KO and *Smox-*KO mice subjected to RLDC point to the important role of polyamine catabolism in the mediation of tubular damage and exacerbation of long term renal injury and fibrosis. 

The potency and anti-neoplastic activity of cisplatin and platinum-derived compounds is reduced due to their off-target toxicity [5,68]. While these toxic effects can contribute to both off-target and anti-neoplastic activities (i.e., injuring both non-dividing parenchymal and actively dividing cancer cells), the formation of stable DNA platinum adducts and interference with cell division are most likely the primary anti-tumor mechanisms of these platinum compounds [2,5,69]. The development of therapeutic approaches that reduce the general harmfulness of these compounds but do not affect their ability to modify DNA structure and inhibit tumor cell proliferation will be a positive step toward improving their therapeutic efficacy. To this end, there are a number of approaches that have already been utilized to reduce their toxicity, such as pre-treatment with agents that increase the susceptibility of cells to lower concentrations of the platinum compound, hydration during pre- and post-treatment periods, or mannitol plus hydration treatment [70]. In addition, molecular pathways that mediate cisplatin nephrotoxicity have been identified [1,71,72]. These include signaling by toll-like receptors (TLRs), activation of programmed necrosis and apoptosis, as well as induction of endoplasmic reticulum stress and the unfolded protein response [1,71,72]. Specific molecules such as NFκB, protein kinase C-δ, TLR, and polyamine catabolic enzymes have been identified as factors that mediate acute high-dose cisplatin nephrotoxicity [1,42,69,73]. The studies presented here identify the role of enhanced polyamine catabolism in the mediation of kidney injury in RLDC. They further demonstrate that the disruption of polyamine catabolism can reduce the severity of fibrosis and long-term renal dysfunction subsequent to treatment with platinum compounds.

## 5. Conclusions

**Figure 10:**
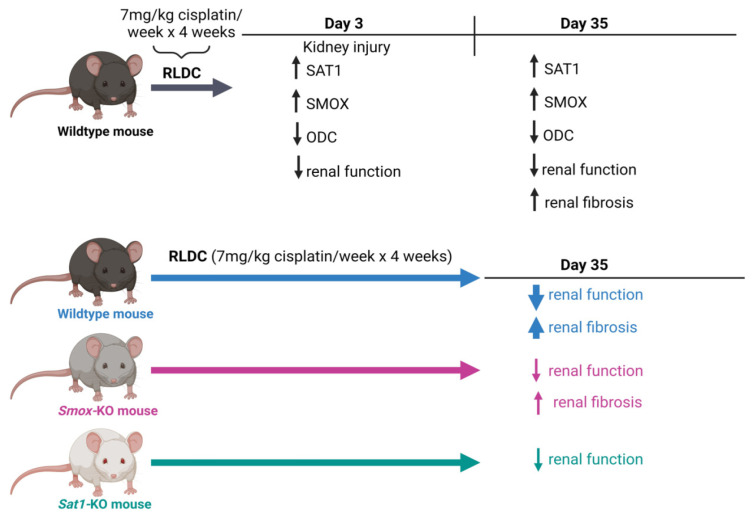
The potential role of polyamine catabolism in RLDC nephrotoxicity. The expression of polyamine catabolic enzymes, SMOX and SAT1, increases, while that of ODC, the first-rate limiting enzyme in polyamine synthesis, decreases in response to RLDC treatment. The ablation of polyamine catabolic enzymes reduces the severity of renal injury, as evidenced by the preservation of renal function and reduced levels of fibrosis in *Sat1*-KO and *Smox*-KO mice. The thickness of the vertical arrows denotes the extent of observed change in renal function and injury.

Based on our current and previous studies [37,42], we propose that increased catabolism of polyamines significantly contributes to the toxic effects and tissue damage caused by cisplatin (Figure 10). Our studies also suggest that treatments that modulate polyamine catabolism or neutralize the toxic by-products of polyamine degradation may be useful in reducing the nephrotoxicity of cisplatin. Such approaches can enhance the therapeutic effectiveness of cisplatin and its derivatives.

## Data Availability

The datasets used and/or analyzed in the current study are included in this published article and its Supplementary Materials. These documents are also available from the corresponding author upon request.

## Supplementary Materials

The following supporting information can be downloaded at: https://www.mdpi.com/article/10.3390/biomedicines12030640/s1, Datasets S1–S4: Day 3 and 35 RNA-seq analyses; Datasets S5–S8: Day 3 and 35 GO Biological Process and Molecular Function Enrichment Analyses; Datasets S9 and S10: Days 3 and 35 KEGG Enrichment analyses; Figure S1: Renal polyamine levels in control and RLDC-treated Wt, *Smox*-KO and *Sat1*-KO mice; Figure S2: Original images of northern blot analysis results.
