# Supplementary material for: Polyamine Catabolism and Its Role in Renal Injury and Fibrosis in Mice Subjected to Repeated Low-Dose Cisplatin Treatment
Source: Biomedicines. 2024 Mar 13;12(3):640. doi: 10.3390/biomedicines12030640 (PMC10968664; doi:10.3390/biomedicines12030640)
Supplement: Supplementary file 1 [file biomedicines-12-00640-s001.zip › Figure S1.pdf]

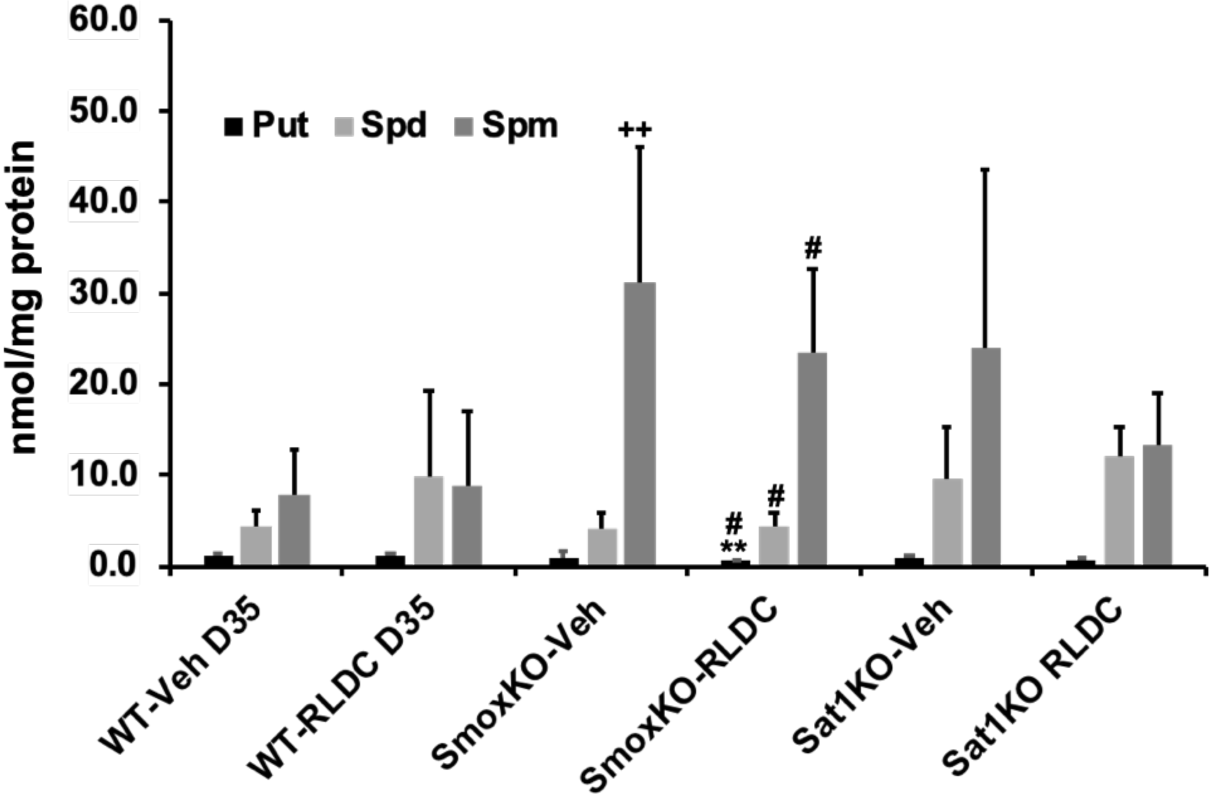

Supplementary Figure S1. Comparison of renal polyamine levels in control and RLDC-treated Wt, *Smox*-KO and *Sat1*-KO mice. Renal polyamine levels were compared on day 35 after the final saline (Veh) or cisplatin (RLDC) treatment. The (**) denotes a p<0.01 when average values of Vehicle and RLDC *Smox*-KO samples were compared. The (**++**) denotes a p<0.01 when average values of Vehicle controls of WT and *Smox*-KO samples were compared. The (**#**) denotes p<0.01 when average values of WT and *Smox*-KO mice subjected to RLDC were compared.
