# Supplementary figures and images for: Polyamine Catabolism and Its Role in Renal Injury and Fibrosis in Mice Subjected to Repeated Low-Dose Cisplatin Treatment
Source: Biomedicines. 2024 Mar 13;12(3):640. doi: 10.3390/biomedicines12030640 (PMC10968664; doi:10.3390/biomedicines12030640)

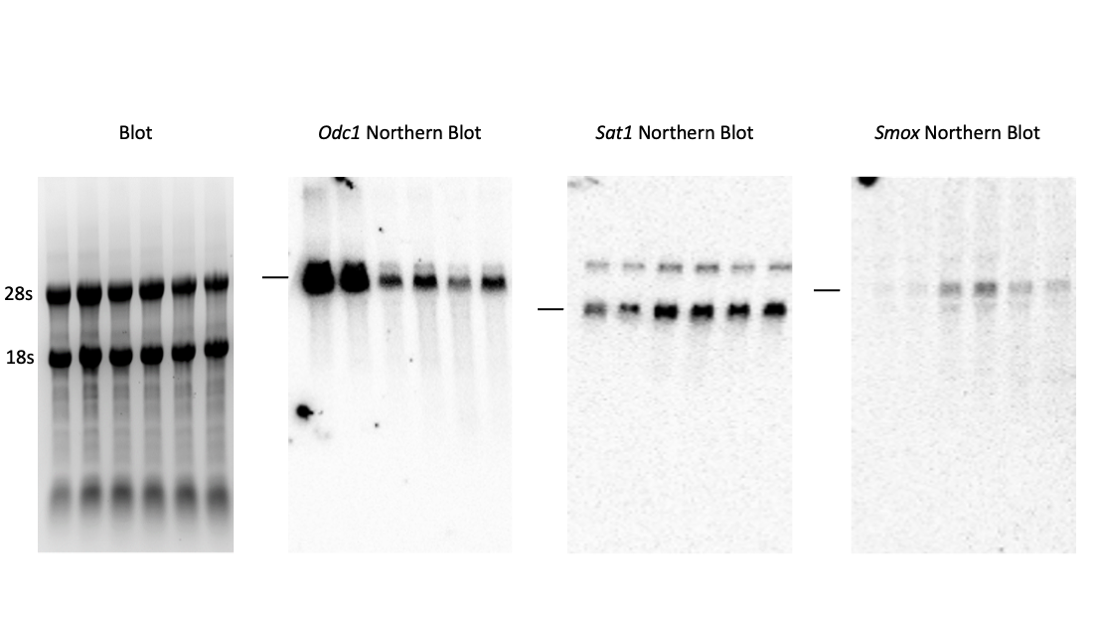

Supplement: Supplementary file 1 [file biomedicines-12-00640-s001.zip › Figure S2.png]
